# Supplementary material for: REAL-TIME VISUALIZATION OF SPLICEOSOME ASSEMBLY REVEALS BASIC PRINCIPLES OF SPLICE SITE SELECTION
Source: bioRxiv. 2024 Jul 13:2024.07.12.603320. Preprint. [Version 1] doi: 10.1101/2024.07.12.603320 (PMC11451613; doi:10.1101/2024.07.12.603320)
Supplement: Supplement 1 [file NIHPP2024.07.12.603320v1-supplement-1.pdf]

bioRxiv preprint doi: https://doi.org/10.1101/2024.07.12.603320; this version posted July 13, 2024. The copyright holder for this preprint (which was not certified by peer review) is the author/funder. This article is a US Government work. It is not subject to copyright under 17 USC 105 and is also made available for use under a CC0 license.

SUPPLEMENTARY FIGURES

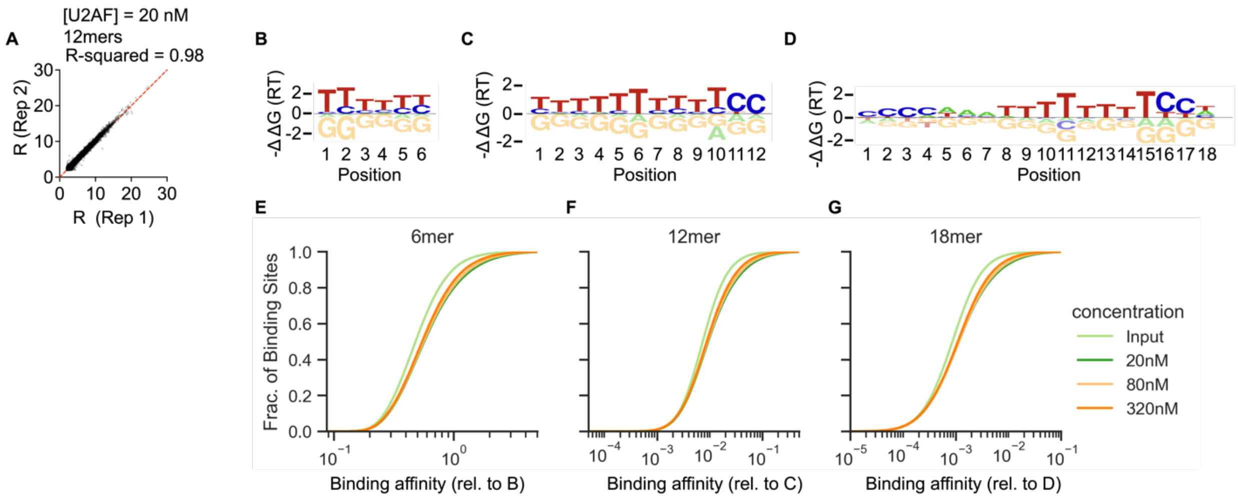

Fig S1. Details on RBNS and Probound Assays

- **A)** RBNS was performed with two replicates of 0, 20, 80, 320 nM U2AF. Comparing enrichment (R) for significantly enriched kmers (Z=3) for U2AF = 20 nM indicates strong agreement between two measurements.
- **B)** U2AF binding model from probound for 6mers.
- **C)** U2AF binding model from probound for 12mers.
- **D)** U2AF binding model from probound for 18mers.
- **E)** Cumulative distribution of predicted binding affinity from the 6mer Probound model for enriched RNA in different replicates of the RBNS assay (input, 20 nM, 80 nM, 320 nM). The RNAs enriched for 20 nM U2AF have the highest binding affinity. Reads from the the initial library (input) have the lowest binding affinity.
- **F)** Same as (E) but for 12mer probound model.
- **G)** Same as (E) but for 18mer probound model.

bioRxiv preprint doi: https://doi.org/10.1101/2024.07.12.603320; this version posted July 13, 2024. The copyright holder for this preprint (which was not certified by peer review) is the author/funder. This article is a US Government work. It is not subject to copyright under 17 USC 105 and is also made available for use under a CC0 license.

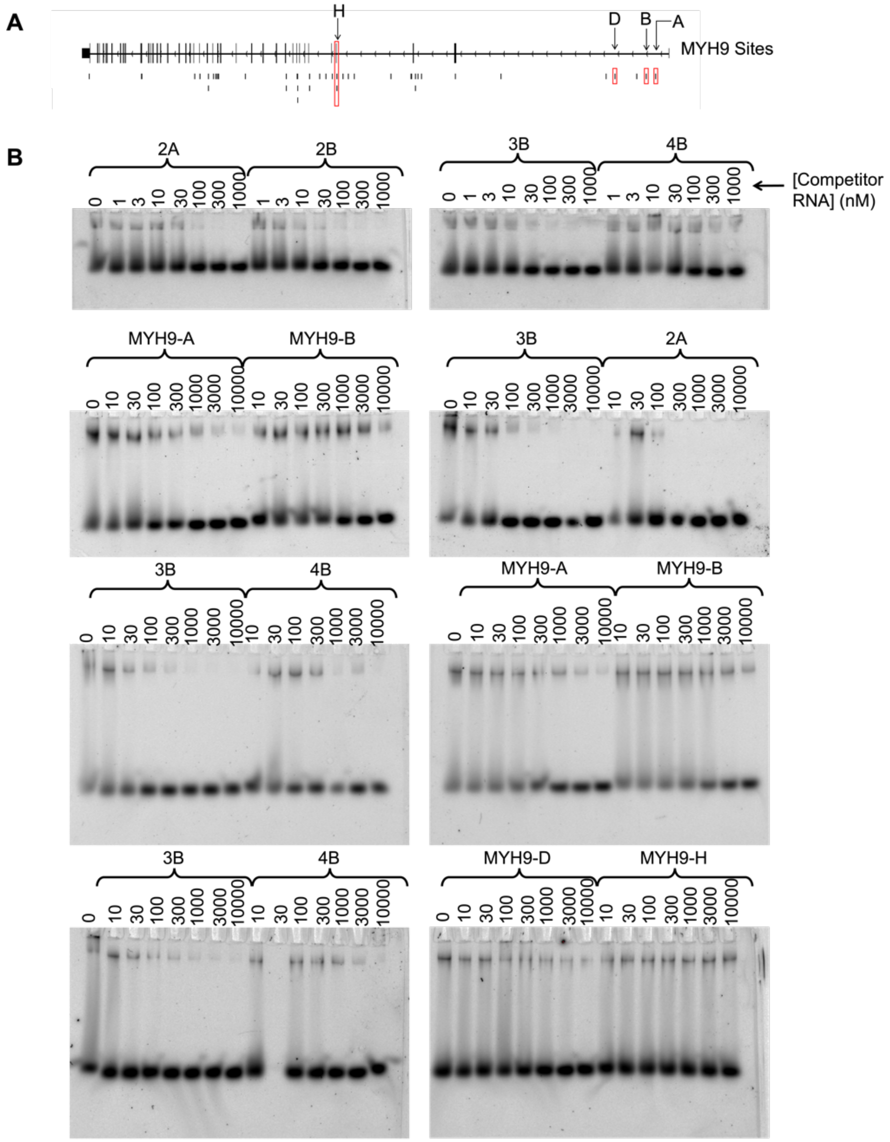

C

	Predicted	Measured IC_{50}		
	1/KD (relative to 3B)	1/ IC_{50} (rel. to 3B)	st dev	replicates
Seq 2A: TTTTTTTTTGGG	0.08743261	0.25574867	0.20484472	2
Seq 2B: TTTTTTTTTCCC	4.56170146	3.63274176		1
**Seq 3B: TTTTTTCCCCCC**	**1**	**1**	**0**	**4**
Seq 4B: TTTCCCCCCCCC	0.03885894	0.03999289	0.03224603	2
MYH9_A: TCCTTTACTTCC	0.37676041	0.15251667	0.04345642	2
MYH9_B: TTAACTCCCTAG	0.02817803	0.00327842	0.00282204	2
MYH9_D: CATCATCTCTCC	0.09671456	0.02563589		1
MYH9_H: TTTCAGACCGAG	0.000358756	No binding		1

Figure S2. Details on Probound validation by competitive binding assay.

- **A)** We tested U2AF binding to various binding sites identified in PAR CLIP in the MYH9 pre-mRNA.
- **B)** EMSA measurements from competitive binding assays.
- **C)** Comparing predicted relative binding affinity from Probound 12mer model with measured U2AF binding affinity from EMSA measurements.

bioRxiv preprint doi: https://doi.org/10.1101/2024.07.12.603320; this version posted July 13, 2024. The copyright holder for this preprint (which was not certified by peer review) is the author/funder. This article is a US Government work. It is not subject to copyright under 17 USC 105 and is also made available for use under a CC0 license.

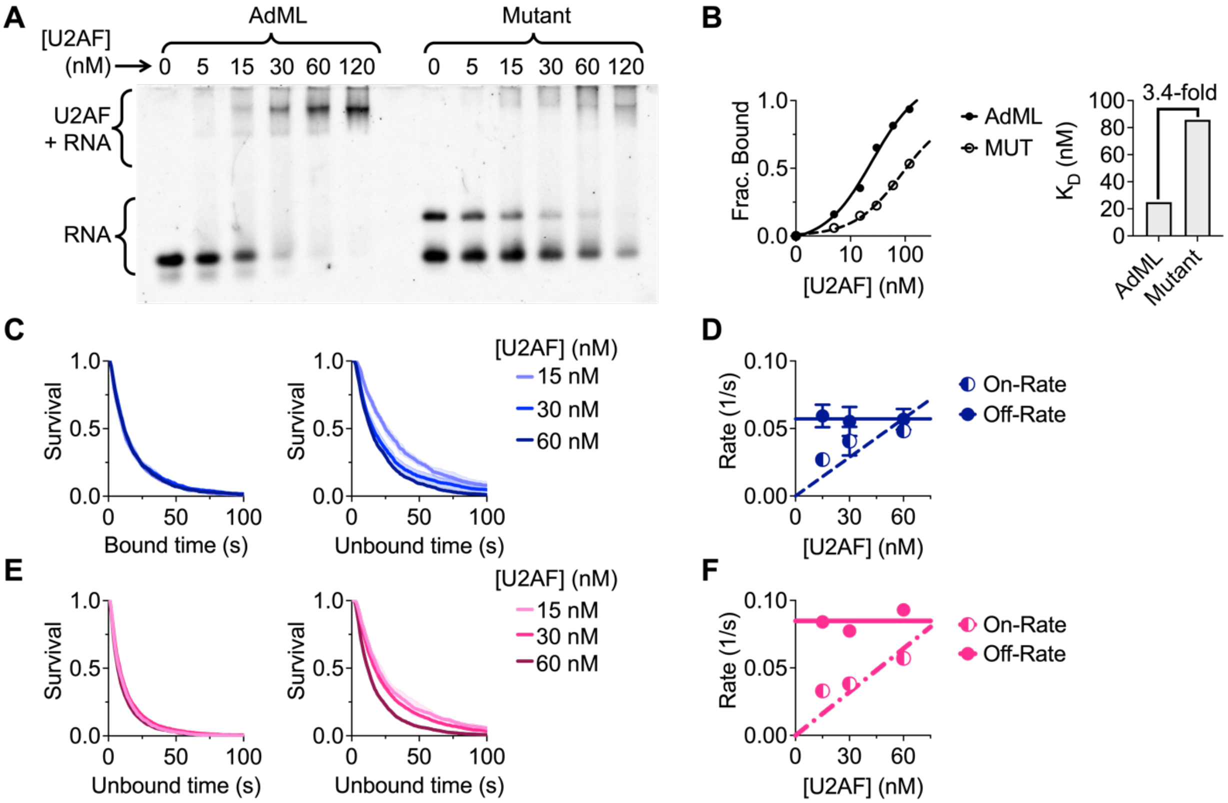

G

RNA	k_{on} (nM^{-1} s^{-1})	k_{off} (s^{-1})
AdML	0.0010 ± 0.0001	0.057 ± 0.003
MUT	0.0011 ± 0.0001	0.085 ± 0.003

Figure S3. Details on in vitro binding assays.

- **A)** Measuring U2AF binding affinity to AdML and Mutant 3'SSs by EMSA.
- **B)** Quantification of EMSA measurement reveals a 3.4-fold difference in binding affinity (n=1 replicate).
- **C)** Dwell time distributions of U2AF in the bound state and unbound state for 3 concentrations of U2AF binding to the AdML 3'SS (n=3 replicates for each concentration).
- **D)** Dwell time distributions were fit to a single exponential distribution to determine the off-rate (from bound time survival plot) and on-rate (from unbound time survival plot) for U2AF binding the AdML 3'SS. This plot shows both rates at each concentration of U2AF. The on-rate is concentration dependent and the off-rate is concentration independent.
- **E)** Same as (C) but for the mutant 3'SS.
- **F)** Same as (D) but for the mutant 3'SS.
- **G)** Table of measured rates of U2AF binding to both 3'SSs.

bioRxiv preprint doi: https://doi.org/10.1101/2024.07.12.603320; this version posted July 13, 2024. The copyright holder for this preprint (which was not certified by peer review) is the author/funder. This article is a US Government work. It is not subject to copyright under 17 USC 105 and is also made available for use under a CC0 license.

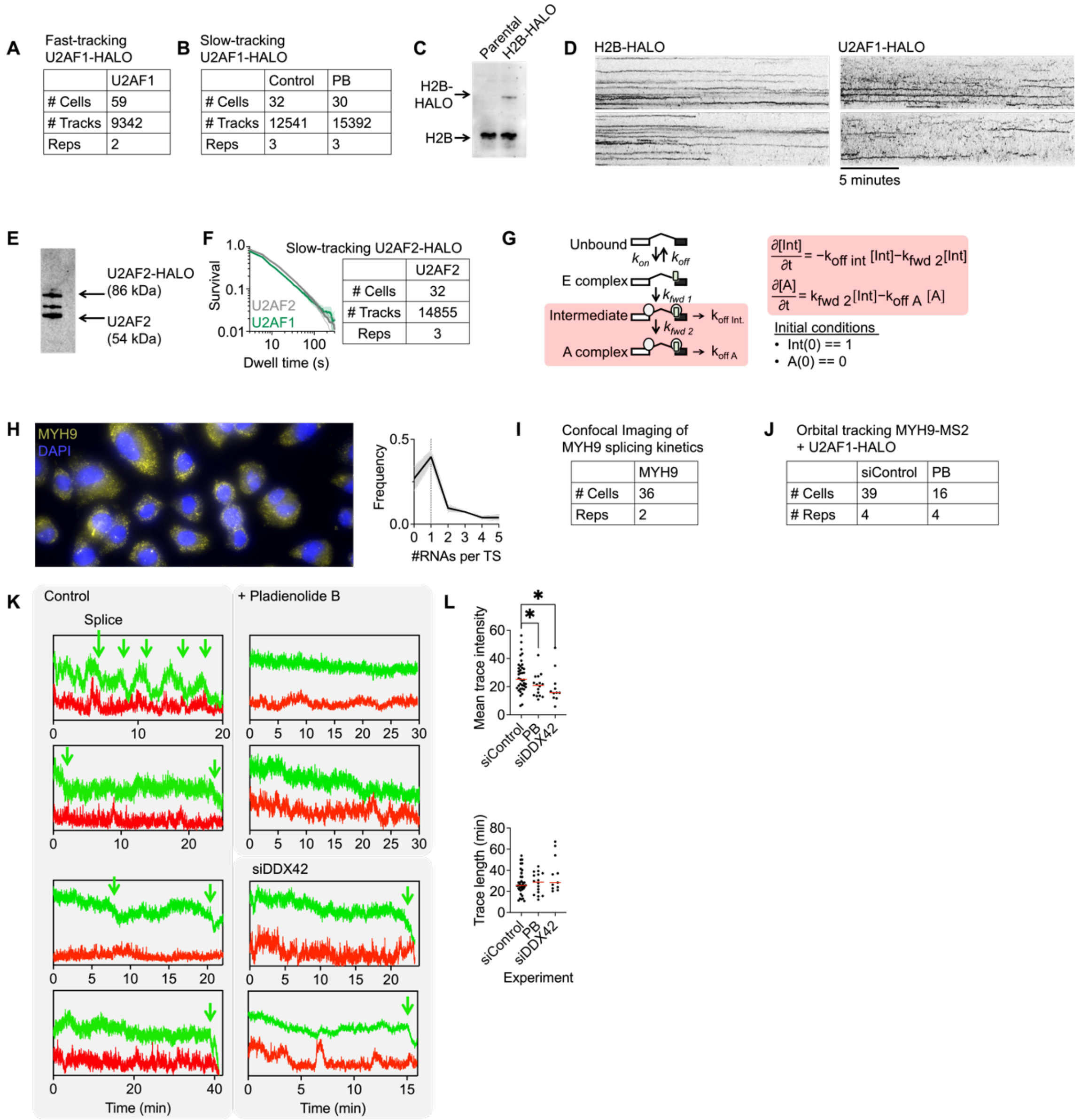

Figure S4. Live cell single-molecule tracking of U2AF in spliceosome assembly.

- ***A)*** Experimental details for fast tracking dataset (100 Hz acquisition rate, 10 seconds) for U2AF1-HALO.
- ***B)*** Experimental details for slow tracking dataset (0.33 Hz acquisition rate, 20 minutes) for U2AF1-HALO (with and without PB treatment).
- ***C)*** Western blot of H2B-HALO HBEC cell line.
- ***D)*** Kymographs of H2B-HALO and U2AF1-HALO in slow tracking conditions.
- ***E)*** Western blot of U2AF2-HALO HBEC cell line.
- ***F)*** Left: Survival plot comparing U2AF1-HALO and U2AF2-HALO dwell times in slow SMT assay. Dwell times appear similar for both cell lines. Right: Experimental details for U2AF2-HALO slow SMT dataset.
- ***G)*** Binding model used to interpret slow SMT data. Equation 1 comes from master equation with initial conditions.

bioRxiv preprint doi: https://doi.org/10.1101/2024.07.12.603320; this version posted July 13, 2024. The copyright holder for this preprint (which was not certified by peer review) is the author/funder. This article is a US Government work. It is not subject to copyright under 17 USC 105 and is also made available for use under a CC0 license.

H) Single-molecule FISH measurements using probes that target the 3'UTR of MYH9. Histogram of number of RNAs per transcription site reveals MYH9 is actively transcribed in many cells.

I) Experimental details for overnight imaging of MYH9 transcription and splicing kinetics.

J) Experimental details for orbital tracking measurements for cells treated with and without pladienolide B (PB).

K) Raw time traces from orbital tracking measurements of MYH9-MS2 (green) and U2AF1-HALO (red). This assays requires U2AF1-HALO to be underlabeled with 5-10 pM JF646 dye. For this reason, while splicing appears in most tracks (green arrows), we did not observe clear U2AF binding in every cell. See methods for details.

L) Plots of mean trace intensity and trace length for each cell analyzed. Generally speaking, transcription sites were followed for more than 20 minutes for all three conditions.

bioRxiv preprint doi: https://doi.org/10.1101/2024.07.12.603320; this version posted July 13, 2024. The copyright holder for this preprint (which was not certified by peer review) is the author/funder. This article is a US Government work. It is not subject to copyright under 17 USC 105 and is also made available for use under a CC0 license.

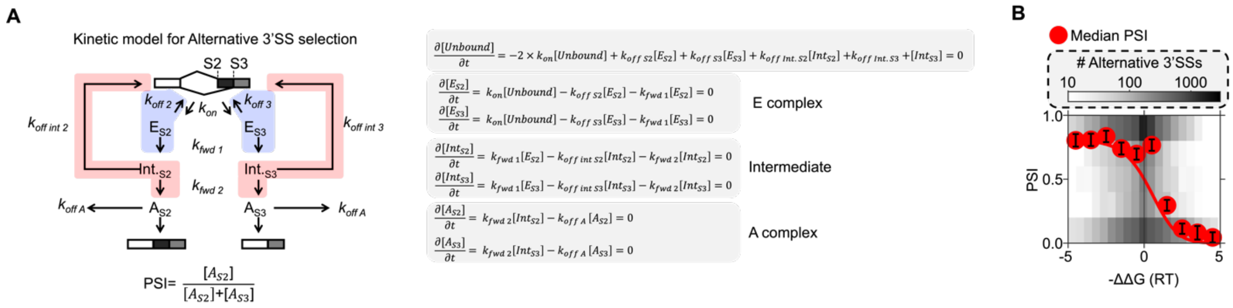

Fig. S5. Alternative splicing model

A) Schematic and master equations for alternative splicing model (equation 2).

B) Plotting median PSI from alternative splicing analysis as in figure 3D. PSI values were binned by −ΔΔG, the free energy of U2AF binding the S3 site relative to the S2 site. Red circles represent median PSI of each bin. Error bars represent the average standard deviation of PSI for each alternative 3'SS (n = 2 replicates). The red curve represents a fit to the alternative splice model (equation 2) (K_s = 0.288 ± 0.001, K_f = 10 (not fit)). To fully describe the variability of this data, we have also included a binned scatter plot showing the underlying variability. The color of each bin corresponds to the number of 3'SSs with contained with a particular −ΔΔG and PSI combination as indicated by scale bar.

bioRxiv preprint doi: https://doi.org/10.1101/2024.07.12.603320; this version posted July 13, 2024. The copyright holder for this preprint (which was not certified by peer review) is the author/funder. This article is a US Government work. It is not subject to copyright under 17 USC 105 and is also made available for use under a CC0 license.

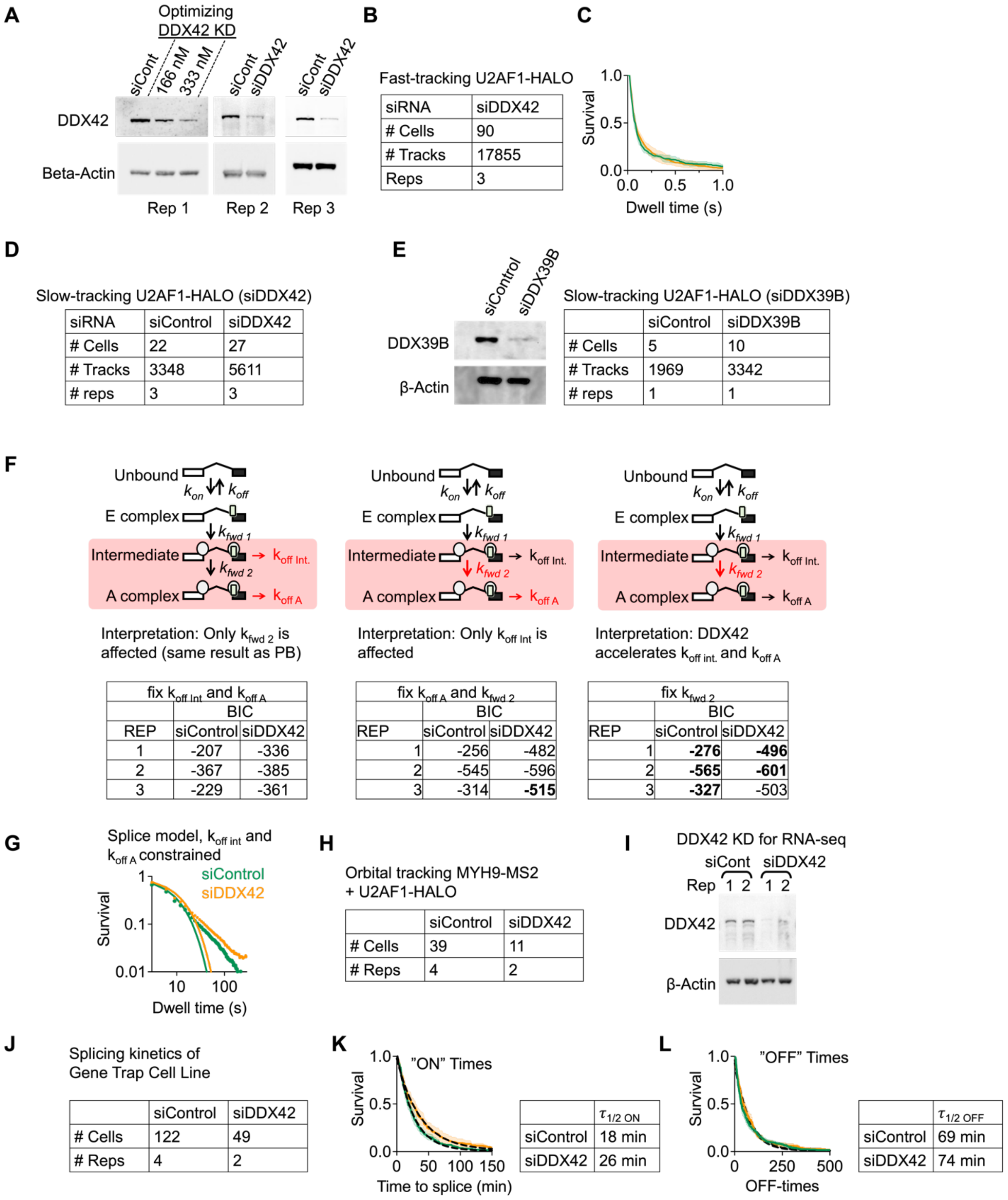

bioRxiv preprint doi: https://doi.org/10.1101/2024.07.12.603320; this version posted July 13, 2024. The copyright holder for this preprint (which was not certified by peer review) is the author/funder. This article is a US Government work. It is not subject to copyright under 17 USC 105 and is also made available for use under a CC0 license.

Figure S6. Characterizing the role of DDX42 in spliceosome assembly kinetics

A) Western blot of DDX42 knockdown for each replicate of the SMT assay
B) Experimental details for fast tracking of U2AF1-halo after DDX42 knockdown.
C) Dwell times times measured in fast SMT assay for siControl and siDDX42 experiments. DDX42 does not seem to impact the U2AF1-HALO dwell times on these time scales.
D) Experimental details for slow tracking of U2AF1-HALO after DDX42 knockdown.
E) Western blot of DDX39B knockodown and experimental details on slow tracking of U2AF1-HALO after DDX39B knockodown.
F) Model testing for simultaneous fitting of siControl and siDDX42 slow SMT datasets. We first tried the exact same fitting conditions as the PB experiment (left panel). Fitting was improved by constraining $k_{fwd\ 2}$ and $k_{off\ A}$ (middle panel) and further improved by only constraining $k_{fwd\ 2}$ (right panel). Results of model testing are shown in table for each replicate. Best fits (as indicated by Bayesian Information Criterion values) are indicated in bold.
G) Simultaneous fit for siControl and siDDX42 slow SMT datasets to the same model as used for PB experiment. We deemed this fit inadequate. BIC values are shown in the left panel of F.
H) Experimental details for orbital tracking experiment (siControl and siDDX42).
I) Western blot of DDX42 knockdown for sample sent for RNA-sequencing.
J) Experimental details for overnight confocal imaging of gene trap cell line.
K) Comparison of ON-time distributions for siControl and siDDX42 in the polyclonal gene trap cell line. ON-time represents the splicing kinetics.
L) Comparison of OFF-time distributions for siControl and siDDX42 from imaging the polyclonal gene trap cell line. 'OFF-time' distribution represents the time between a splicing event and the start of a transcription event.
